# Intramammary metastatic melanoma of unknown primary origin in a 58-year old patient: a case report

**DOI:** 10.1186/s13256-016-1117-y

**Published:** 2016-12-20

**Authors:** Zeina El-Tani, Christophe Duc, Thomas Gluecker, Olivier Cottier

**Affiliations:** 1 Service de Gynécologie-Obstétrique, HRC (Hôpital Riviera-Chablais), Aigle, Switzerland; 2 Institut d’Histocytopathologie, ICHV, Sion, Switzerland; 3 Service de Radiodiagnostic et de Radiologie, HRC Suisse, Aigle, Switzerland

**Keywords:** Breast cancer, Melanoma, Metastatic, Diagnosis, Therapy

## Abstract

**Background:**

Malignant melanoma metastasis to the breast is a rare disease.

**Case presentation:**

We present the case of a 58-year-old postmenopausal Caucasian woman with metastatic malignant melanoma of unknown origin of the right breast. She presented with a palpable lump in the inferior quadrant of her right breast. The investigations concluded it was breast metastasis from a malignant melanoma of unknown origin. The treatment consisted of mastectomy and axillary lymph node dissection. Two lymph nodes were positive for tumor cells and one showed extracapsular extension. Our patient did not receive immediate adjuvant therapy. Six weeks after the surgery, our patient presented a relapse in the right axilla (a 6 × 4 cm mass) with positive internal mammary lymph nodes and a single brain metastasis. This relapse motivated an adjuvant treatment with partial regression of the disease. Currently, our patient presents multiple metastases with poor prognosis.

**Conclusions:**

From this experience, we advocate an immediate aggressive handling of melanoma metastasis to the breast.

## Background

The worldwide incidence and mortality rate of malignant melanoma have been constantly increasing over the past 50 years in fair-skinned populations. Melanoma is the fourth most frequent cancer in Switzerland. Its incidence rate in Switzerland is one of the highest in Europe with 24.6 out of 100,000 [1]. Approximately 20% of malignant melanomas will metastasize, whether by hematogenic or lymphatic route. The breast is a rare site of metastases for extramammary tumors (incidence 1.3–2.7%) [2]. Malignant melanoma is the most common cancer to metastasize to the breast. Therefore, in patients with a history of malignant melanoma, the possibility of a metastasis should be included in the differential diagnosis.

Establishing the diagnosis can be difficult. Clinical examination and imaging techniques are not specific. Cytological and pathological examinations, with the help of immunohistochemical stainings, are the key to the diagnosis. In most cases, treatment consists of surgical resection. The need for chemo-, radio- or immunotherapy is case-dependent.

Finally, we would like to underline that this disease can have a very aggressive course, as was the case with our patient.

## Case presentation

We present here the case of a 58-year-old postmenopausal Caucasian woman with metastatic malignant melanoma of unknown origin of the right breast. The patient came to our emergency department in December 2014 after noticing a lump in her right breast. Upon physical examination, a well-circumscribed mass of 2 cm was confirmed in the lower external quadrant with no skin involvement and no enlarged lymph nodes. A thorough examination of the skin revealed no other lesion. The patient had no previous history of malignant melanoma or of removal of suspect skin lesion.

**Fig. 1:**
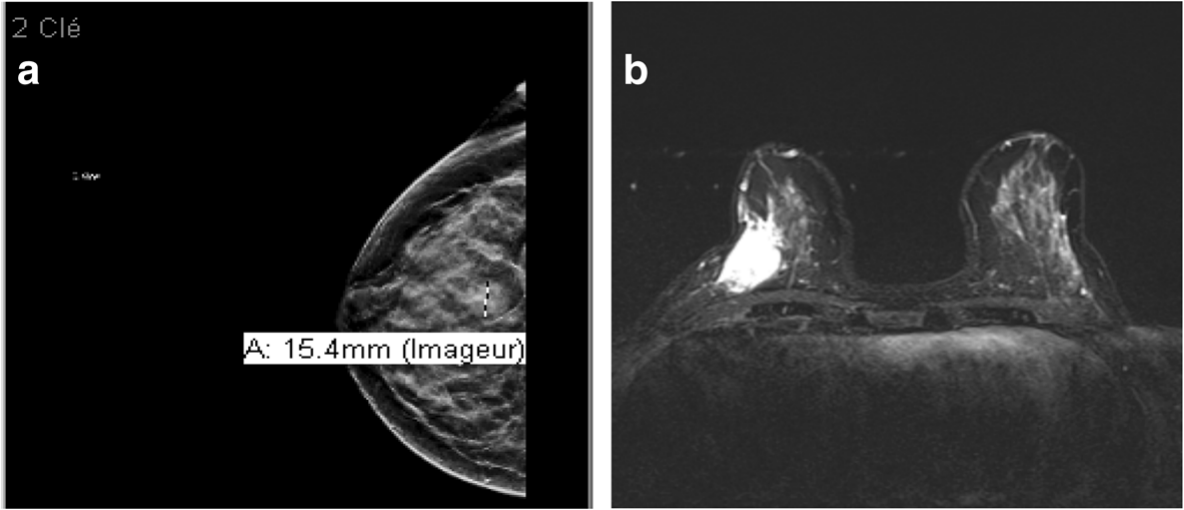
**a** Initial mammography. Well-delimited mass in the lower external quadrant of the right breast. **b** Initial magnetic resonance imaging. Mass in the lower external quadrant of 2.8 × 3.0 × 3.0 cm. From the subcutaneous plane to the pectoral plane with no infiltration of the pectoral muscle. Central necrosis with peripheral contrast enhancement

The mammography and sonography examinations showed an oval 16 × 10 × 13 mm, well-delimited hypervascular mass, parallel to the skin (Fig. 1a). Our patient underwent sonographically guided core biopsies. The microscopic examination showed a poorly differentiated tumor with medium to large cells with eosinophilic cytoplasm and pleomorphic nuclei. The immunohistochemistry evaluation was negative for estrogen and progesterone receptors, as well as for E-cadherin and HER-2. It was positive for protein S-100 and vimentin, therefore compatible with the diagnosis of malignant melanoma. The KI-67 was 100%.

A magnetic resonance imaging (MRI) scan revealed a 3 cm lesion with no cutaneous or pectoral infiltration (Fig. 1b) and a positron emission tomography-computed tomography (PET-CT) scan showed a hypermetabolic mammary mass with a homolateral metastatic axillary adenopathy.

The case was presented to the multidisciplinary tumor board of our referent tertiary center. It was decided to repeat the biopsies in order to confirm the diagnosis. Thus, our patient underwent a second biopsy of the breast lesion and of the axillary adenopathy (5 February 2015) seen on the PET-CT scan. The diagnosis of metastatic malignant melanoma of unknown origin with positive axillary ipsilateral adenopathies was confirmed.

**Fig. 2:**
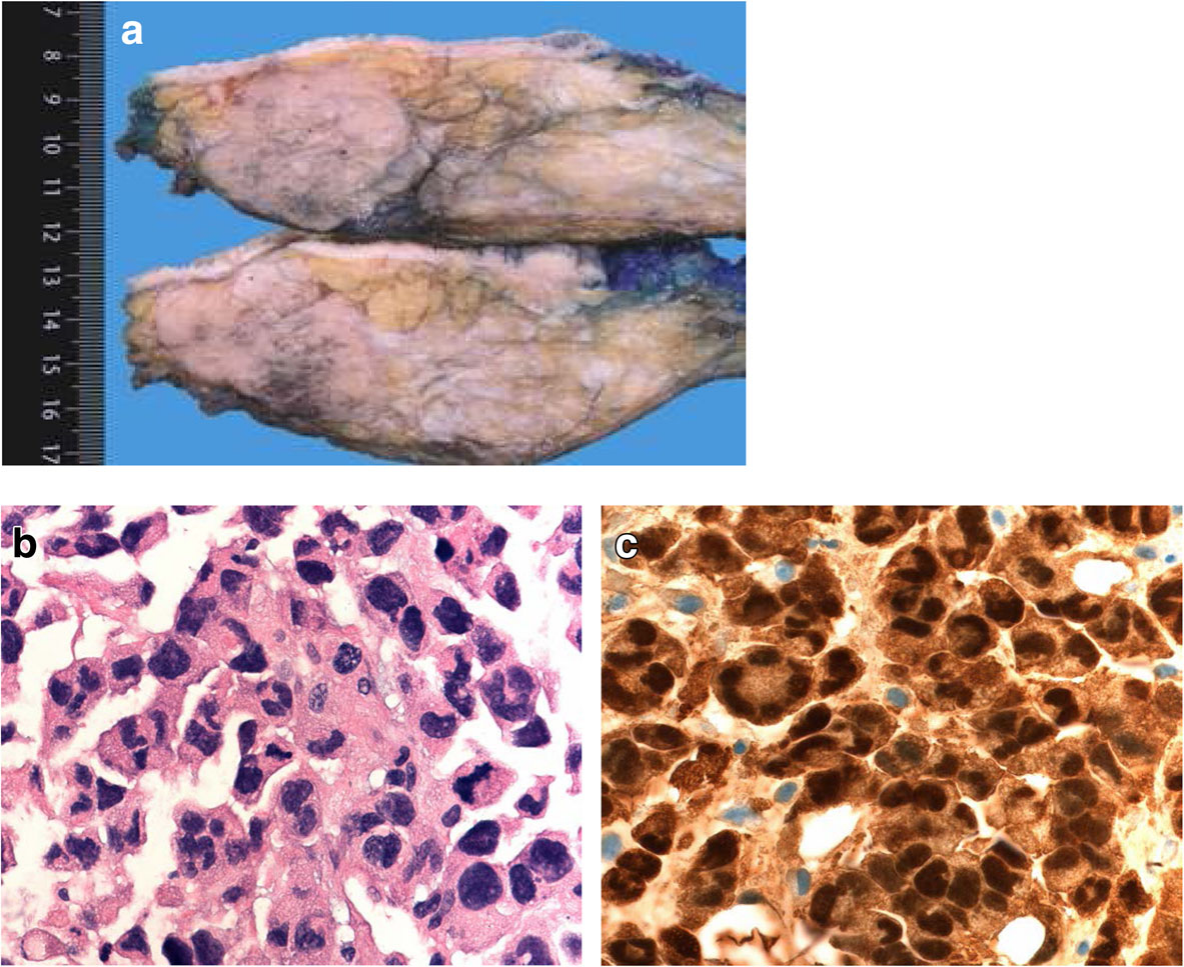
**a** Macroscopy of the tumor. The cut section of the breast tumor is nodular, tan to gray and more or less well-demarcated. **b** Standard histology. The tumor cells are very anisokaryotic with abundant eosinophilic cytoplasm and numerous often atypical mitoses (hematoxylin and eosin (H&E), ×40). **c** Immunohistochemistry. All tumor cells are strongly immunoreactive for S-100 in the cytoplasm and in the nuclei (S-100, ×40)

Our patient underwent a mastectomy with axillary lymph node dissection in March 2015. The histopathological examination found a 4.5 × 4 × 3.9 cm mass, with tumor-free margins: 0.4 cm of the cranial plane and 0.35 cm of the deep plane (Fig. 2a). The tumor cells were of medium size with hyperchromatic nuclei and anisokaryosis. Some of the cells showed a large and eosinophilic cytoplasm. The immunohistochemistry examination was positive for protein S-100 and negative for Melan-A and HMB-45. Other immunohistochemical markers were tested for and were negative: epithelial markers (pancytokeratin, Ber-EP4, p63, keratin 903, keratin 5/6, keratin 8/18), muscular markers (actin, desmin, caldesmon), lymphohistiocytic markers (CD68 [KP1], PGM1, CD1a, CD4, CD43, CD45), endothelial markers (CD31 and CD34). The c-Kit was also negative (Fig. 2b and c).

Molecular biology sequencing showed no mutations for *KRAS* gene (exons 2-5), *NRAS* gene (exons 2-5) and *BRAF* gene (exon 15).

From the axillary lymph node dissection, 17 lymph nodes were excised, of which two were positive for tumor cells and one presented extracapsular extension.

Our patient underwent a complete dermatological, ophthalmological, ENT, gynecological and gastroenterological (colonoscopy and gastroscopy) examination without finding a primary lesion. A cerebral MRI scan did not show any primary lesion or metastases. Our patient did not receive adjuvant therapy, according to the decision of the multidisciplinary tumor board of the referent tertiary center.

**Fig. 3:**
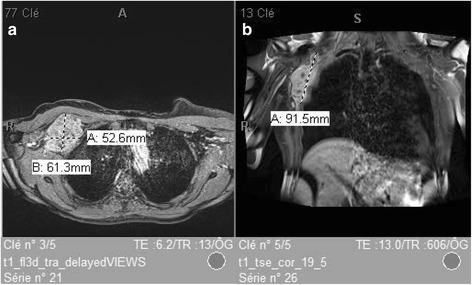
**a** and **b** Relapse magnetic resonance imaging. Right axillary mass in the right pectoralis minor muscle, in contact with the thoracic wall. Satellite nodule in contact with the superior and inferior poles. Tumoral nodule in the mastectomy site. Right axillary and internal mammary adenopathies

Six weeks later, our patient presented with a palpable mass in the right axilla. At the sonographic examination, a hypervascular 6 × 4 cm mass was visualized with a satellite nodule of 1.6 × 1.2 cm, both of which were biopsied. The results showed the same histologic and immunohistochemical characteristics of the breast tumor, with a diffuse nuclear and cytoplasmic S-100 expression. An MRI scan (28 May 2015) (Fig. 3a and b) confirmed the right axillary polylobulated 9 × 5 × 7 cm mass, in the pectoralis minor muscle with thoracic wall infiltration, as well as a satellite nodule of 2.7 cm and a single brain metastasis (Fig. 3c). A complementary PET-CT scan (3 June 2015), showed a hypercaptation at the site of the mastectomy with invasion of the pectoralis major muscle, as well as a hypercaptation in the right axilla and in the internal mammary lymph nodes. There was no hepatic, pulmonary, adrenal, or osseous dissemination

Our patient was referred to the tertiary center, and mid-June, a treatment combining stereotaxic irradiation of the brain metastasis and adjuvant therapy with ipilumimab was initiated. At the end of August, the control cerebral MRI and PET-CT scans showed the partial regression of the brain metastasis. The right axillary mass was decreased in size.

## Discussion

Approximately 20% of malignant melanomas will metastasize, whether by hematogenous or lymphatic route. The incidence of breast metastases from extramammary tumors varies between 1.3 and 2.7% [2]. According to a review of the literature by Koch *et al*. [3], malignant melanoma is the most common cancer to metastasize to the breast (29.8%). Other cancers known to metastasize to the breast are: lung cancer (16.4%), gynecological cancers with a majority of ovarian cancers (12.7%), digestive tumors (9.9%), leukemia and lymphomas (8.4%), sarcomas (7.3%), and renal tumors (1.5%). Malignant melanoma can also occur as a primary intramammary tumor [4].

In 70% of the cases, the patients are premenopausal [2]. The lesions are mostly found in the upper external quadrant [5]. In this particular situation, our patient was a postmenopausal woman and the lesion was situated in the inferior external quadrant.

### Diagnosis

The diagnosis of malignant melanoma of the breast can be challenging. The sonographic and mammographic findings of metastatic nodules are very diverse and cannot be used to differentiate between a metastasis and a primary mammary adenocarcinoma. A metastatic nodule may even mimic a benign lesion radiographically. It has been reported in the literature that on mammography, metastatic nodules appear as well defined opacities without calcifications. Sonographically, nodules are well-defined, round or oval, and hypoechogenic with a well-defined posterior wall [5, 6]. The only finding that can differentiate it from a benign nodule is the increased vascularity of the lesion [7], as in this situation.

The key element in establishing the diagnosis of malignant melanoma of the breast is the histopathological examination combined with immunohistochemistry staining techniques. The melanoma cells’ appearance and architectural disposition are very diverse. Hematoxylin and eosin (H&E) sections cannot be complete without immunohistochemistry. No immunohistochemistry marker is 100% specific or sensitive. The protein S-100 is a very sensitive marker for melanoma (expressed in 95% of tumors) but it is not specific and should be used in combination with others markers such as Melan-A, HMB-45 and tyrosinase, which are much more specific (present in 70% of melanomas). Moreover, melanomas can express other markers such as CD31, CD68, epithelial membrane antigen, and CAM5.2 [8]. In this case, the markers expressed were protein S-100 and vimentin.

### Staging and prognosis of melanoma and metastatic melanoma of unknown primary origin (MUP)

Staging of melanoma in this particular case is challenging since the tumor presented itself as a melanoma metastasis as well as positive lymph nodes without a detectable primary lesion. Regarding the anatomic stage groupings for cutaneous melanoma (clinical and pathologic staging), this case should be considered at least a stage III (Any T, ≥N1, M0), or even a stage IV (Any T, any N, M1; M1a: metastases to skin, subcutaneous, or distant lymph nodes) [9, 10]. By convention, the anatomic staging should be used after complete excision of the primary lesion, which was not feasible in this situation, since the primary lesion was unknown.

An ongoing debate exists in the literature regarding the difference in prognosis when comparing metastatic melanoma of unknown primary origin (MUP) and metastatic melanoma of known primary origin (MKP) [11]. Some studies show a better prognosis when the primary lesion is unknown (with the same corresponding tumor stage) [12]. Other authors conclude that MUP patients with nodal metastases have a similar survival, compared with MKP stage III patients with macroscopic involvement, and that MUP patients with distant metastases have a similar survival as MK stage IV patients [13].

As mentioned before, in MUP patients, the staging is difficult as it is hard to distinguish which patient has a regional or a distant (sub)cutaneous or nodal metastasis. Therefore, the prognosis is difficult to establish [13]. Nonetheless, the management of MUP patients should be the same as those with stage-matched MKP [11].

### Surgical treatment

The main treatment of malignant melanoma is wide excision with free margins combined with sentinel node biopsy. Lymph node resection should also be performed if axillary node involvement is positive. Mastectomy and internal mammary node dissection are not recommended currently [4].

Our patient underwent mastectomy and axillary node dissection as a positive lymph node was confirmed by biopsy.

### Adjuvant radiotherapy (RT)

There is no consensus on the use of adjuvant radiotherapy after lymph node dissection in malignant melanoma, but it has been recently shown in a phase III trial that RT could decrease the rate of local recurrence, following surgery. The trial showed, however, that there was no impact on overall survival. There was a reduction of 52% of lymph node field relapse in patients who had undergone radiotherapy, but no differences for the relapse-free survival (70 vs. 73 relapses, hazard ratio [HR] 0.91, 95% confidence interval [CI] 0.65–1.26; *p* = 0.56) or overall survival (59 vs. 47 deaths, HR 1.37, 95% CI 0.94–2.01; *p* = 0.12).

In our case, the patient had extranodal spread and did not receive adjuvant radiotherapy. A lymph-node field relapse occurred 6 weeks after the surgery with a mass of 9 × 5 × 7cm with a satellite nodule of 2.7 cm [14].

### INF-α, chemotherapy and immune vaccines

INF (interferon)-α is the standard of care in selected high-risk patients with stage III [9]. High doses of INF-α 2b have been proved, in clinical trials, to have a beneficial effect on distant disease-free survival, but not on overall survival [15]. The mechanism by which it targets melanoma cells is not fully understood. It has been shown to downregulate MEK/ERK MAPK, an important pathway of cell metastasis as well as STAT3, another pathway for cell survival, metastasis, proliferation, angiogenesis, and immune evasion.

Chemotherapy has not shown any beneficial effect on either distant-free survival or overall survival, whether it is used as a single agent or in combination with other chemotherapeutics, hormonal or biological therapy. Chemotherapeutic agents include dacarbazine and hydroxyurea [15].

Immune vaccines tend to activate an antitumor response by cytotoxic T cell response. In the case of melanoma, specific melanoma antigens are incorporated (MART1/Melan-A, gp100, tyrosinase), which should be potentially recognized by cytotoxic T lymphocytes. The patients who responded to any of the peptides and developed a T cell response showed survival times that were doubled compared to those who did not respond. Other antigens have been evaluated with no survival benefits, some are even suspected to have induced immunosuppression [16].

### New therapies

A better understanding of the immune system modulation and of the genetic mutations underlying melanoma cells has led to the development of new agents.

These agents attack melanomas through two different pathways: (1) by modulating the immune system to target melanoma cells or (2) by altering the cell cycle of melanoma cells with oncogene-targeted therapies [17, 18].

Through immune-modulating antibodies (anti-CTLA4, anti-PD1, anti-CD40, anti-CD137, and anti-OX40), an antitumor immune response is created. One of those immune-modulating antibodies is the anti-CTLA4 antibody ipilimumab (IgG1). The CTL4 antigen is a checkpoint that downregulates T cell activation and proliferation. The antibody targets, and therefore blocks, the CTL4 antigen, resulting in an upregulation of T cell activation and proliferation.

Two phase III studies showed the superiority of this treatment compared to dacarbazine and peptide vaccines. Ipilimumab reduces the risk of recurrence by 20% with an overall survival that is stable and sustainable at 3 years [17, 18]. On the other hand, targeted therapies block specific pathways or specific mutated oncogenes. Known oncogenic activating mutations are: BRAF, c-KIT and NRAS.

BRAF, for example, is known for its activating mutation V600E, an important actor for the proliferation of melanoma cells. Vemurafenib is a BRAF inhibitor that offers overall and progression-free survival [19]. It has been approved by the Food & Drug Agency (FDA) for the treatment of unresectable metastatic melanoma. Unfortunately, although the initial response is very high, the tumor develops mechanisms of resistance against these drugs. Instead, multiple pathways should be targeted in order to achieve a successful therapy [18].

It seems that the molecular status of the melanoma at the time of the histological diagnosis needs to be determined, so that each patient can be treated individually according to the specificities of the melanoma [18].

## Conclusions

Malignant melanoma of the breast, whether primary or metastatic, is a particularly rare and aggressive disease. It is of primary importance to make an early diagnosis and immunohistochemistry plays a major role. The treatment consists of adequate resection. Adjuvant therapy is not mandatory, but should be started without delay, if found necessary, as relapse can occur in a very short period of time.

This case has shown a very aggressive course of the disease with fast locoregional relapse and emergence of distant metastases. It outlines the difficulty in the assessment of the need of adjuvant therapy. From this experience, we advocate an immediate aggressive handling of melanoma metastasis to the breast.

## Abbreviations

HER: Human epidermal growth factor receptor
FDA: Food & Drug Agency
H&E: Hematoxylin and eosin
ENT: Ear, Nose and Throat
INF: Interferon
